# GRP78 translocation to the cell surface and O-GlcNAcylation of VE-Cadherin contribute to ER stress-mediated endothelial permeability

**DOI:** 10.1038/s41598-019-47246-w

**Published:** 2019-07-25

**Authors:** Raji Lenin, Peter G. Nagy, Kumar Abhiram Jha, Rajashekhar Gangaraju

**Affiliations:** 1 Department of Ophthalmology, University of Tennessee Health Science Center, Memphis, TN USA; 2 Department of Anatomy & Neurobiology, University of Tennessee Health Science Center, Memphis, TN USA

**Keywords:** Retina, Glycosylation

## Abstract

Increased O-GlcNAcylation, a well-known post-translational modification of proteins causally linked to various detrimental cellular functions in pathological conditions including diabetic retinopathy (DR). Previously we have shown that endothelial activation induced by inflammation and hyperglycemia results in the endoplasmic reticulum (ER) stress-mediated intercellular junction alterations accompanied by visual deficits in a tie2-TNF-α transgenic mouse model. In this study, we tested the hypothesis that increased ER stress via O-GlcNAcylation of VE-Cadherin likely contribute to endothelial permeability. We show that ER stress leads to GRP78 translocation to the plasma membrane, increased O-GlcNAcylation of proteins, particularly VE-Cadherin resulting in a defective complex partnering leading to the loss of retinal endothelial barrier integrity and increased transendothelial migration of monocytes. We further show an association of GRP78 with the VE-Cadherin under these conditions. Interestingly, cells exposed to ER stress inhibitor, tauroursodeoxycholic acid partially mitigated all these effects. Our findings suggest an essential role for ER stress and O-GlcNAcylation in altering the endothelial barrier function and reveal a potential therapeutic target in the treatment of DR.

## Introduction

Endoplasmic reticulum (ER) is the first organelle of the secretory pathway that maintains proper folding and secretion of proteins essential for cell survival^1^. Perturbations of ER function by various pathological conditions like inflammation, hyperglycemia, calcium flux, hyper/hypo glycosylation of proteins and phosphorylation state of the unfolded protein response (UPR) signaling proteins, including accumulation of unfolded or misfolded proteins, results in ER stress^2^. Some of these misfolded proteins or proteins that are glycosylated may escape from the ER to other locations of the cell and display differential expression of surface glycosylation central to a variety of pathological conditions^3,4^.

Glycosylation of plasma membrane and secretory proteins as a post-translational modification (PTM) is a global phenomenon and more than 50% of the proteins in nature are glycosylated^5,6^. The glycosylation pathways occur in the cytosol, ER, and the Golgi complex and involve in the transportation and processing of glycosidases, and glycosyltransferases, the essential players of glycosylation^7^. O-linked glycosylation is one of the most common PTM, involving the addition of N-acetyl-glucosamine (O-GlcNAc) to serine/threonine residues. Given that O-GlcNAc is globally increased in the cultured cells and animal models of diabetes and in humans^8,9^, it is plausible that O-GlcNAcylation plays a key a role in hyperglycemia-induced tissue-damage. O-GlcNAcylated proteins are observed in every part of the eye including the lens, cornea, retinal pigment epithelium, and the neural retina^10,11^. The pathologic development of retinal diseases and vision impairment is associated with compounding risk factors including oxidative stress, inflammation, and ER stress^12^. Specifically, several pathways including vascular endothelial growth factor (VEGF), tumor necrosis factor (TNF)-α, platelet-derived growth factor (PDGF)B, the kallikrein-kinin system, advanced glycation end products, and the polyol pathway were shown to be involved in the pathophysiology of diabetic retinopathy (DR)^13^. Many of these pathways are inter-related, and some of the pathways may potentially involve altered O-GlcNAc signaling. The complex interdependence of ER stress and glycosylation during protein quality control and cellular response to pathophysiological stress remains to be studied.

During ER stress, glucose-regulated protein 78 (GRP78) dissociates from the ER transducers [inositol-requiring enzyme 1α (IRE1α), PKR- like ER kinase (PERK) and activating transcription factor 6 (ATF6)] to activate the cellular response to the unfolded proteins. GRP78 that reside inside the ER in physiological conditions translocate to the cell surface under pathological conditions as observed in certain type of cancers^14,15^ and autoimmune diseases^16^. The ER resident chaperones including GRP78 are tagged with the peptide KDEL (Lys-Asp-Glu-Leu) at their carboxyl-terminal as an ER retention signal^17^. Importantly, when these proteins escape from the ER to Golgi, KDEL receptors (KDELR) recognize the KDEL-tagged proteins and package them back to the ER. It has recently been shown that during ER stress, loss of KDELR1 or KDEL peptide leads to GRP78 escape from the ER to the cytosol, plasma membrane or extracellular space contributing to various pathologies^18^. More importantly, the cell surface GRP78 then functions as a signaling molecule and may play an important role in the regulation of the pro-proliferative/ anti-apoptotic and pro-migratory signaling pathways^19,20^.

Endothelium under physiological conditions is quiescent, and its integrity is maintained by tight and adherens junction (AJ) proteins that perform a variety of cellular functions^21^. AJ proteins maintain cell-cell adhesion, intracellular signaling and transcriptional regulation via calcium-dependent homophilic adhesion between cells and the actin cytoskeleton. Cadherins and catenins represent AJ proteins. Among the cadherins, vascular endothelial cadherin (VE-Cadherin) being the major player has a vital role in vascular endothelial integrity^22,23^. Under certain pathophysiological situations such as in diabetes, endothelial cells have been shown to be activated resulting in a variety of PTM of junction components which ultimately affect protein stability and binding interactions that will lead to increased vascular permeability^21^.

Recently, we demonstrated that endothelial activation induced by inflammation and hyperglycemia results in ER stress-mediated intercellular junction alterations accompanied by visual deficits in an endothelial specific tie2-transmembrane TNF-α transgenic mouse model^24^. To better understand the molecular mechanism(s) behind these events, in this study we tested the hypothesis that during ER stress, loss of KDELR1 leads to GRP78 translocation, increased glycosylation of junction proteins and subsequent loss of retinal endothelial barrier integrity. Using our well-established *in vitro* model of human retinal endothelial cells exposed to a combination of TNF-α and high glucose, we demonstrate that ER stress activation results in increased GRP78 translocation to the plasma membrane. Increased ER stress correlated with augmented O-GlcNAcylation of proteins, in particular, VE-Cadherin. Subsequently, we show that increased O-GlcNAcylation lead to loss of retinal endothelial barrier integrity and increased transendothelial migration of monocytes. Interestingly, translocated GRP78 is found to be associated with VE-Cadherin. Finally, ER stress inhibitor (Tauroursodeoxycholic acid, TUDCA) could ameliorate the aforementioned events albeit partially.

## Results

### ER stress activation in HREC cells

**Figure 1:**
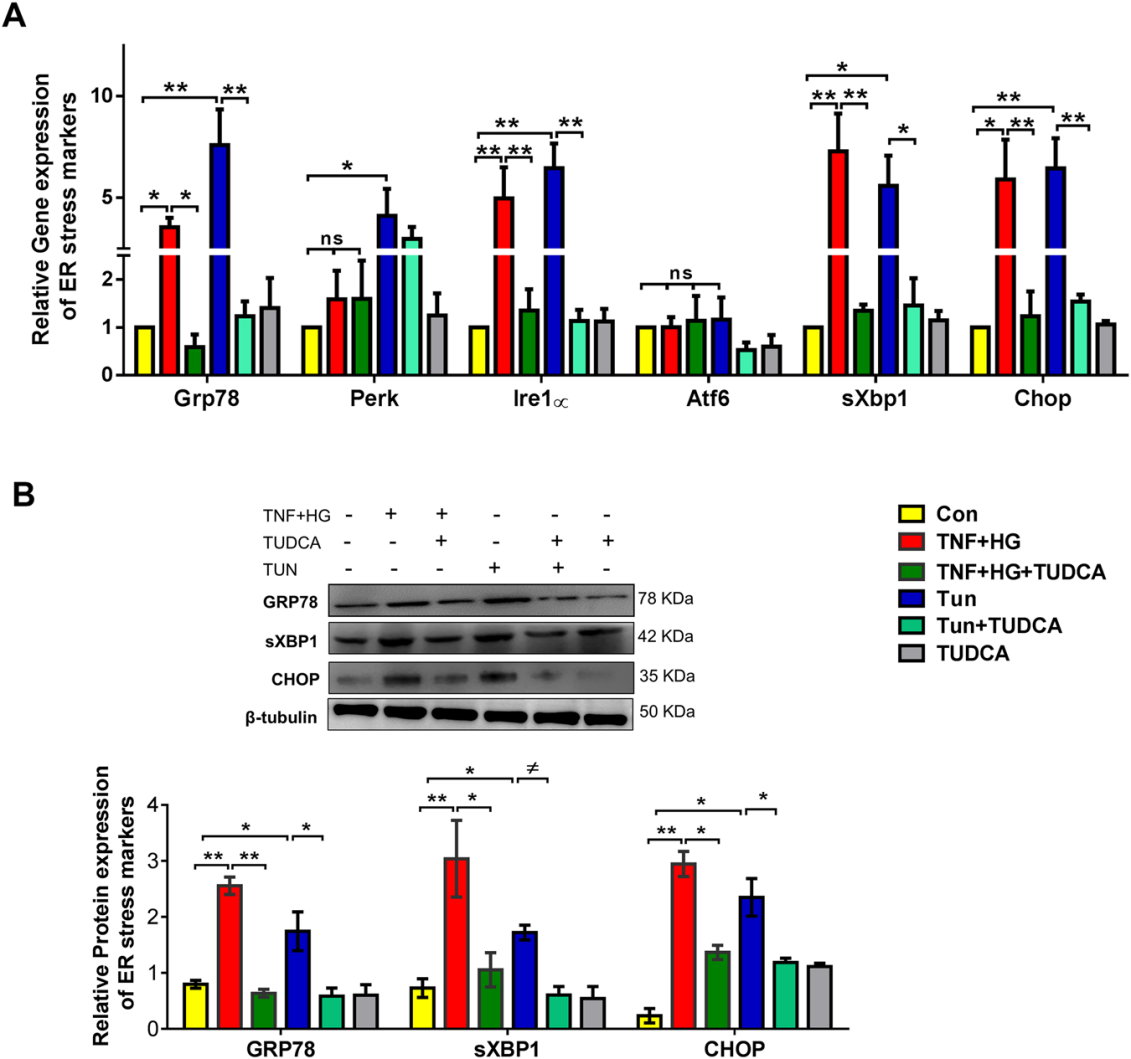
HRECs exposed to TNF-α, and high glucose for 24 h demonstrated increased ER stress. Gene expression (**A**) and protein expression (**B**) of ER stress markers with and without TUDCA. Tunicamycin (Tun) served as positive control for ER stress. 18S rRNA served as an internal control for gene expression and β-tubulin for protein expression studies. Data represent Mean ± SEM from 3 independent experiments performed in duplicates. **p < 0.01; *p < 0.05; ^#^p > 0.05.

We first asked whether HRECs exposed to TNF-α (TNF) and high glucose (HG) for 24 h elicit ER stress response. HRECs treated with either TNF-α alone or HG alone did not show any appreciable increase in ER stress markers while the combination demonstrated a robust 3-fold increase in ER stress markers (Sup. Fig. 1). HRECs exposed to TNF + HG increased gene expression of Grp78, Ire-1α, spliced X box binding protein (sXbp-1) and C/EBP homologous protein (Chop) (3–6-fold, p < 0.05; Fig. 1A) and increased protein expression of GRP78, sXBP-1 and CHOP (2–3-fold, p < 0.05; Fig. 1B) when compared to unstimulated cells. However, gene expression of PERK and ATF6 were unaffected. On the other hand, HRECs pre-treated with TUDCA and exposed to TNF + HG significantly abrogated the increase in the above ER stress markers (p < 0.05). Tunicamycin, a known ER stress activator in UPR pathway exhibited up to 7-fold (p < 0.05) increase in gene and up to 2-fold (p < 0.05) increase in protein expression of ER stress markers served as a positive control in the assay. Neither HRECs pre-treated with TUDCA nor the cells exposed to L-Glucose alone did not affect the above markers.

### GRP78 translocation to the plasma membrane

**Figure 2:**
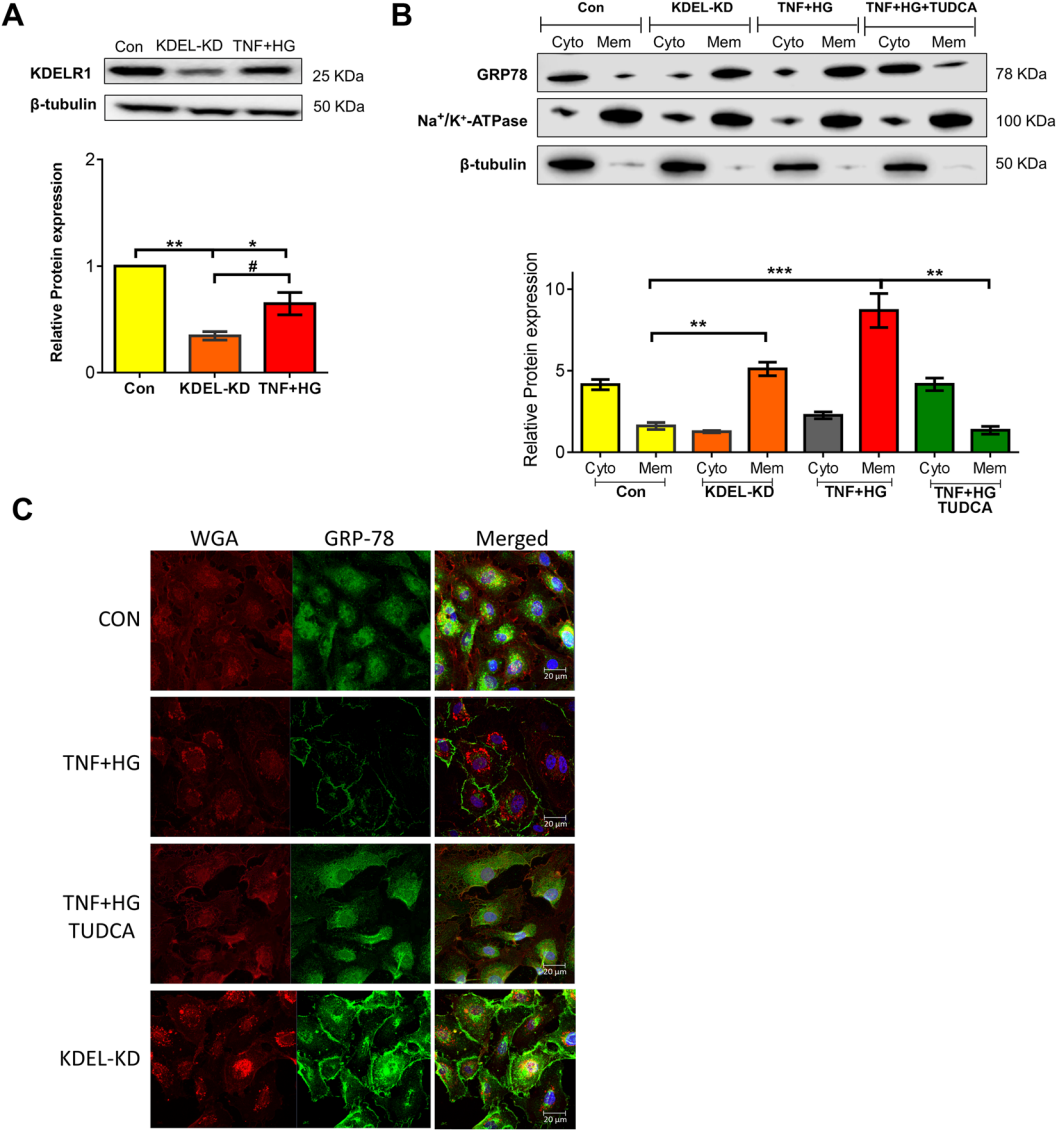
HRECs exposed to TNF-α, and high glucose for 24 h demonstrated increased GRP78 translocation. HRECs silenced for KDELR1 (KDEL-KD), confirmed with protein expression of KDELR1 in total cell lysates (**A**). GRP78 protein expression in cytosolic (Cyto) and membrane (Mem) fractions from various treatments. Na^+^/K^+^-ATPase served as an internal control for membrane fraction, while β-tubulin represented a cytosolic fraction (**B**). Representative confocal immunofluorescence images of the cells stained for GRP78 and WGA (**C**). Data represent Mean ± SEM from 3 independent experiments performed in duplicates. ***p < 0.001; **p < 0.01; *p < 0.05, ^#^p > 0.05.

Since we observed an increase in the levels of GRP78, we postulated that GRP78 might exceed the retention capacity of KDEL retrieval system, resulting in its escape from ER to the cell surface^18^. To address this possibility, we silenced the cells for KDELR1. Silencing was confirmed by protein expression of KDELR1 in the total cell lysate. KDELR1 expression was significantly reduced in KDELR1 knockdown (KDEL-KD) cells compared to control cells. Cells treated with TNF + HG showed a similar reduction in KDELR1 (p < 0.05) (Fig. 2A). Cytosolic and membrane fractions obtained from various treatments were then analyzed for GRP78 expression. Interestingly, KDEL-KD cells demonstrated a significant increase in GRP78 in membrane fraction with little to no expression in the cytosol fraction. Notably, a similar marked expression of GRP78 was observed in the membrane fractions from TNF + HG treatment. Conversely, pre-incubation of HRECs with TUDCA demonstrated significant amelioration of the observed translocation effects with TNF + HG (p < 0.05) (Fig. 2B). To further confirm the membrane translocation of GRP78, immunocytochemistry followed by confocal microscopy was employed. While HRECs treated with TNF + HG showed staining of GRP78 mostly in the membrane, cells that were silenced with KDELR1 siRNA also demonstrated similar membrane expression of GRP78. In line with other studies, HRECs treated with TUDCA showed an inhibition of membrane translocation (Fig. 2C).

### Total O-GlcNAcylation profile of HRECs

**Figure 3:**
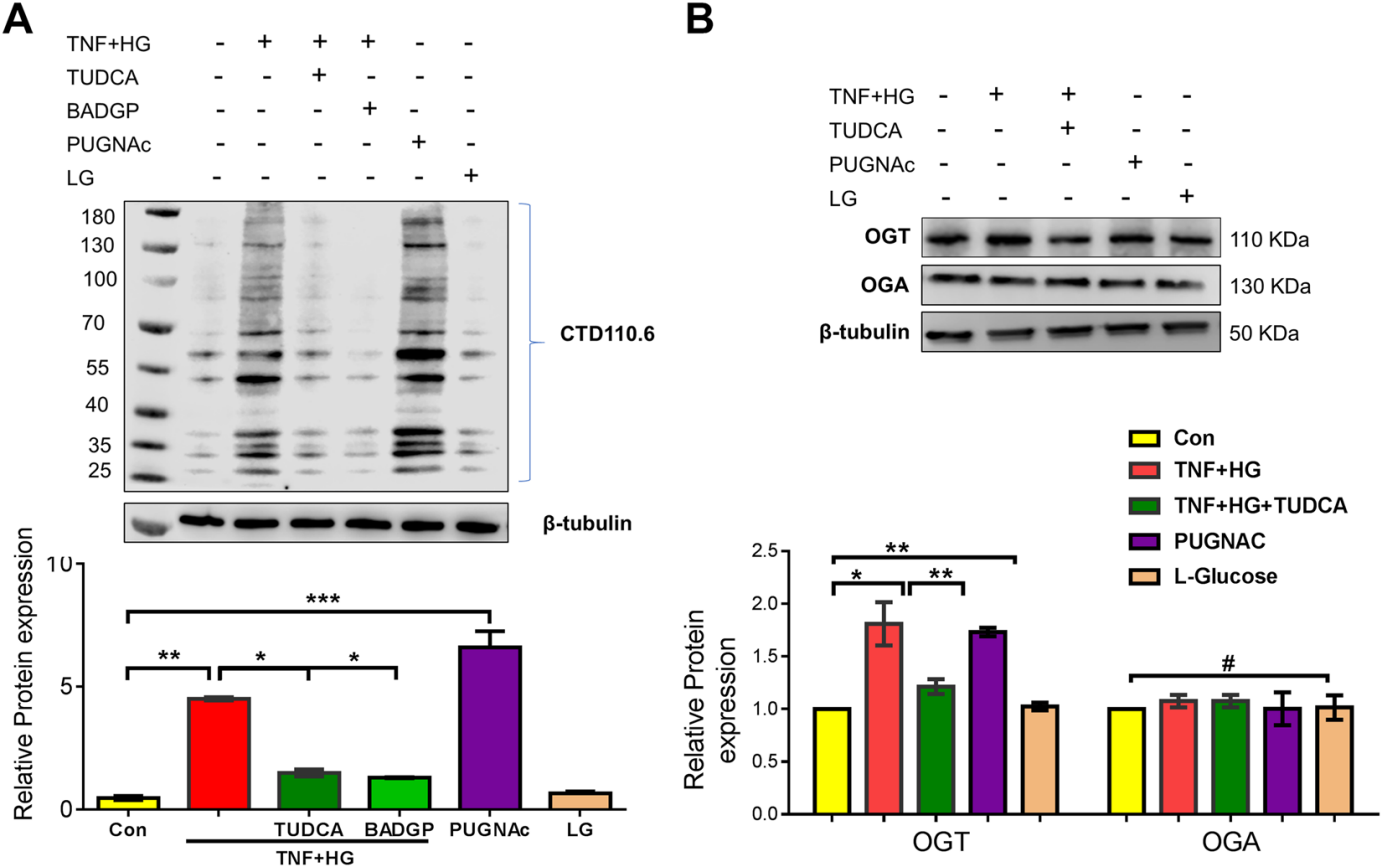
HRECs exposed to TNF-α, and high glucose for 24 h demonstrated increased total O-GlcNAcylation. O-GlcNAcylation confirmed by protein expression (clone: CTD.116.0) (**A**), protein expression of O-glycosyl transferase (OGT) and O-GlcNAcase (OGA) enzyme (**B**). HRECs treated with PUGNAc served as positive control. β-tubulin served as an internal control. Data represent Mean ± SEM from 3 independent experiments performed in duplicates. ***p < 0.001; **p < 0.01; *p < 0.05, ^#^p > 0.05.

Followed by the observation of GRP78 translocation, we postulated changes in O-GlcNAcylation, a major PTM implicated under ER stress. To this end, we investigated the alterations in O-GlcNAcylated protein levels in HRECs treated with TNF + HG using the immunoblotting technique. While HRECs treated with TNF + HG probed with anti-O-GlcNAc (clone CTD110.6) antibody exhibited a significant increase in total proteins that were O-GlcNAcylated, HRECs pre-treated with TUDCA significantly inhibited the increase in O-GlcNAcylation (p < 0.05; Fig. 3A). To address which specific proteins are glycosylated, we performed Glycosylation Antibody Array studies to simultaneously detect the glycosylation protein profile with and without TUDCA after TNF + HG treatment in HRECs. Our analysis demonstrated about 70 proteins found to be significantly upregulated (>1.5 fold vs. ctrl) or downregulated (0.65 fold vs. ctrl; Sup. Fig. 2 & Sup. Table 1). Of these, Focal adhesion kinase, a well-known player of vascular permeability^25^ is 2.3 fold increased in TNF + HG while TUDCA treated cells showed a decrease (0.88 fold vs. TNF + HG). Similarly, Cathepsin D, a well-known protein implicated in endothelial permeability^26^ upregulated by about 1.8 fold in TNF + HG with a significant decrease with TUDCA treatment (0.98 fold).

To confirm O-GlcNAcylation is involved in our studies, we exposed HRECs to PUGNAc, an activator for glycosylation, which served as a positive control with greater than a 5-fold increase in total O-GlcNAcylation levels by immunoblotting. Additionally, HRECs pre-treated with BADGP, an O-glycosylation inhibitor and challenged with TNF + HG showed reduced expression of O-GlcNAcylated proteins. Since O-GlcNAcylation regulated by O-GlcNAc-transferase (OGT) and O-GlcNAcase (OGA) enzymes, we next checked for the expression of these proteins in HREC lysates. While OGT significantly increased in HRECs treated with TNF + HG compared to untreated cells, OGA levels remained unchanged. Finally, cells exposed to TUDCA and challenged with TNF + HG demonstrated a significant reduction in OGT, while those cells exposed to PUGNAc showed a 2-fold increase in OGT levels, which is in line with the observed total O-GlcNAcylation levels (Fig. 3B).

### Transendothelial migration of monocytes across HRECs

**Figure 4:**
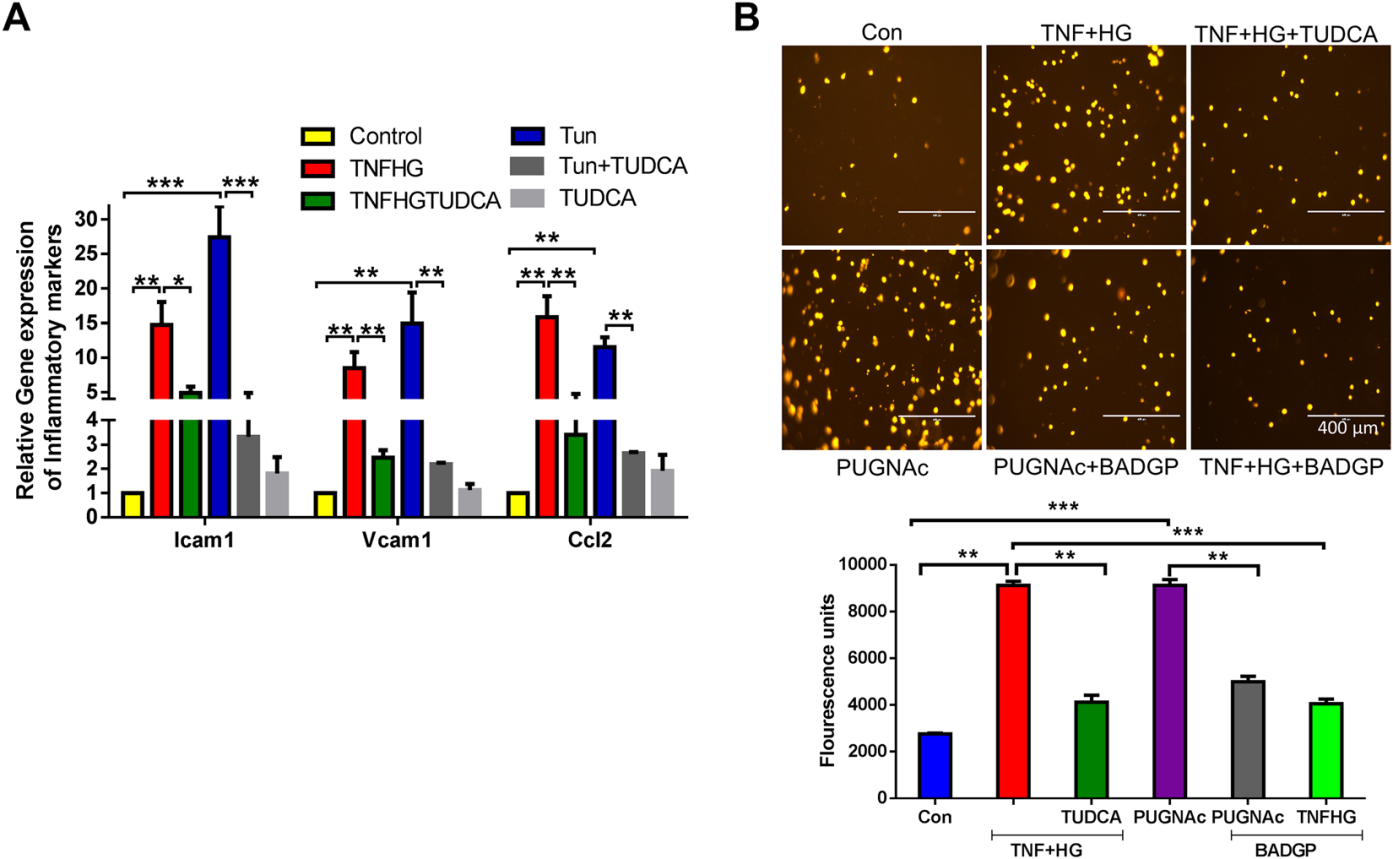
HRECs exposed to TNF-α, and high glucose for 24 h demonstrated increased transmigration of monocytes. Gene expression of inflammatory markers (**A**). Representative images of transmigrated leukocytes from the respective wells. Quantification of fluorescence intensities from various treatments (**B**). Data represent Mean ± SEM from 3 independent experiments performed in triplicates. ***p < 0.001; **p < 0.01. Scale bar = 400 μm.

Previously we have shown that transmigration of activated leukocytes across endothelial cells as a functional readout of compromised endothelium^27^. First we tested if TNF + HG induced changes in endothelial adhesion and chemokine proteins in our model. To this end we show a >5-fold increase in Icam1, Vcam1 and Ccl2 gene expression suggesting activated endothelium (Fig. 4A). To confirm if O-GlcNAcylation is involved in the compromised endothelium, we tested HRECs exposed to TNF + HG with and without inhibitors. The human monocytic leukemia cell line THP-1 exhibited only a low spontaneous migration through an endothelial monolayer of control cells, which dramatically increased in TNF + HG treated endothelial cells. On the other hand, TUDCA was able to reverse these observations. Interestingly, while HRECs incubated with PUGNAc also resulted in increased transendothelial migration of THP-1 cells, those HRECs treated with BADGP showed a reduction (Fig. 4B).

### O-GlcNAcylation mediated vascular permeability defects

**Figure 5:**
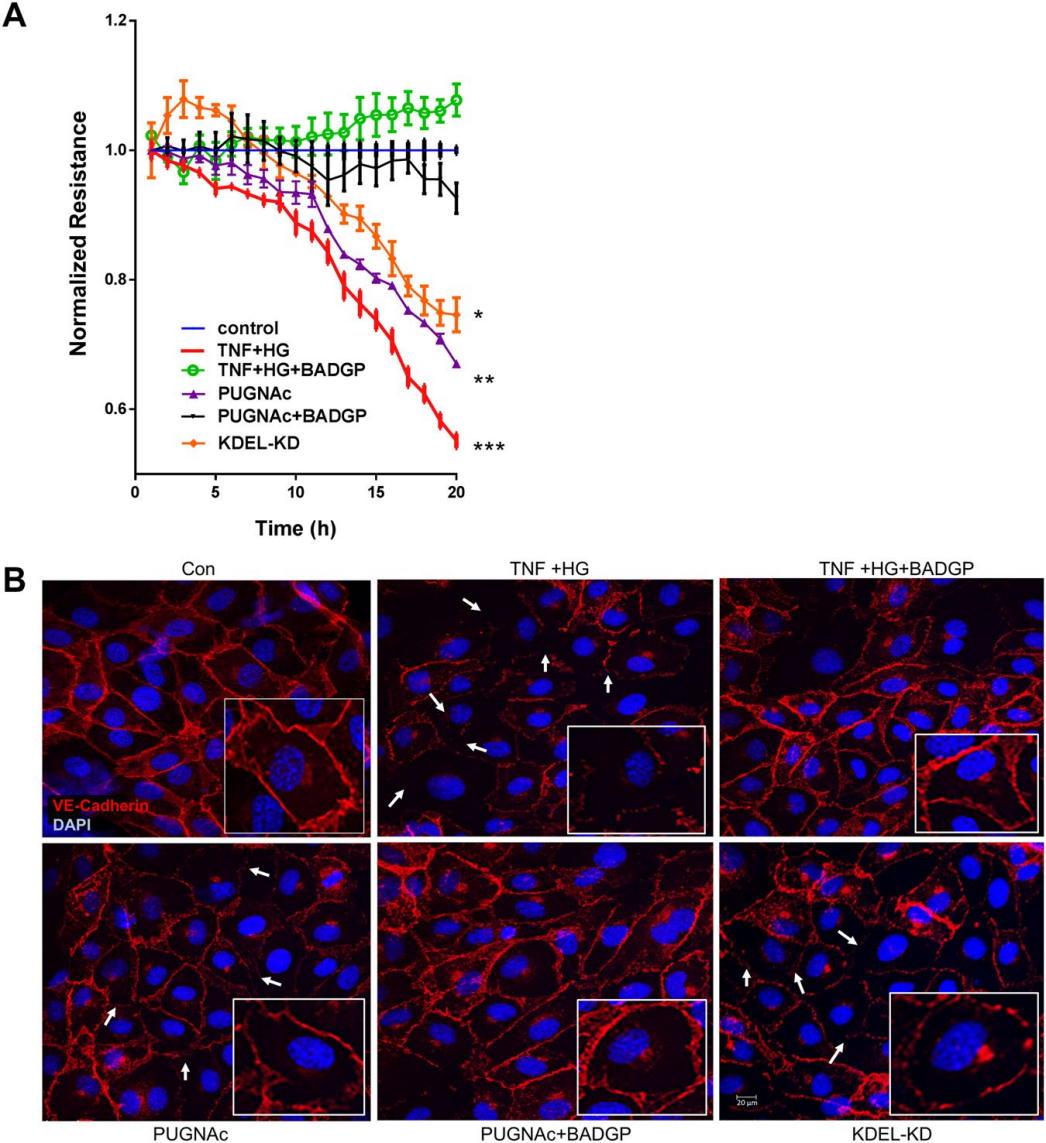
HRECs exposed to TNF-α, and high glucose for 24 h demonstrated decreased trans-endothelial electrical resistance. Representative ECIS tracings plotted as normalized resistance from all treatment groups (**A**). Representative confocal immunofluorescence images of the cells stained for VE-Cadherin. Boxed images shows higher magnification. White arrows indicate loss of cell-cell contacts (**B**). Data represent Mean ± SEM from 3 independent experiments performed in duplicates. **p < 0.01; *p < 0.05 compared to controls. Scale bar = 20 μm.

Previously we have shown that HRECs exposed to TNF + HG induced ER stress with a sustained reduction in barrier integrity as evidenced by decreased TER^24^. Based on this, we next determined if the observed decrease in TER is mediated by increased O-GlcNAcylation of proteins. For this, HRECs were exposed to PUGNAc. HRECs exposed to PUGNAc induced a sustained reduction in barrier integrity as evidenced by decreased TER compared to untreated control cells (PUGNAc, 0.63 ± 0.01; control, 1.0 ± 0.0 AU, p < 0.05). On the other hand, these effects were rescued by treatment with BADGP (0.94 ± 0.03 A.U., p < 0.05), suggesting that endothelial permeability defects are indeed mediated through O-GlcNAcylation. We then tested if KDELR1 silencing in cells exhibits any permeability defects. As expected, KDEL-KD cells (treated with KDELR1 siRNA) demonstrated a sustained reduction in barrier integrity as compared to negative control siRNA treated cells, albeit at lower levels than in HRECs exposed to TNF + HG (KDEL-KD, 0.74 ± 0.01; control, 1.0 ± 0.0 AU, p < 0.05; TNF + HG, 0.51 ± 0.01 AU, p < 0.05) (Fig. 5A). We have previously reported that the alterations in TER observed in TNF + HG treated endothelial cells coincided with a change in VE-Cadherin distribution. As expected, control cells displayed a continuous pattern of VE-Cadherin staining while those cells exposed to PUGNAc showed partial loss of VE-Cadherin expression that could be reversed by BADGP treatment (Fig. 5B).

### O-GlcNAcylation of VE-Cadherin and its partners in complex

**Figure 6:**
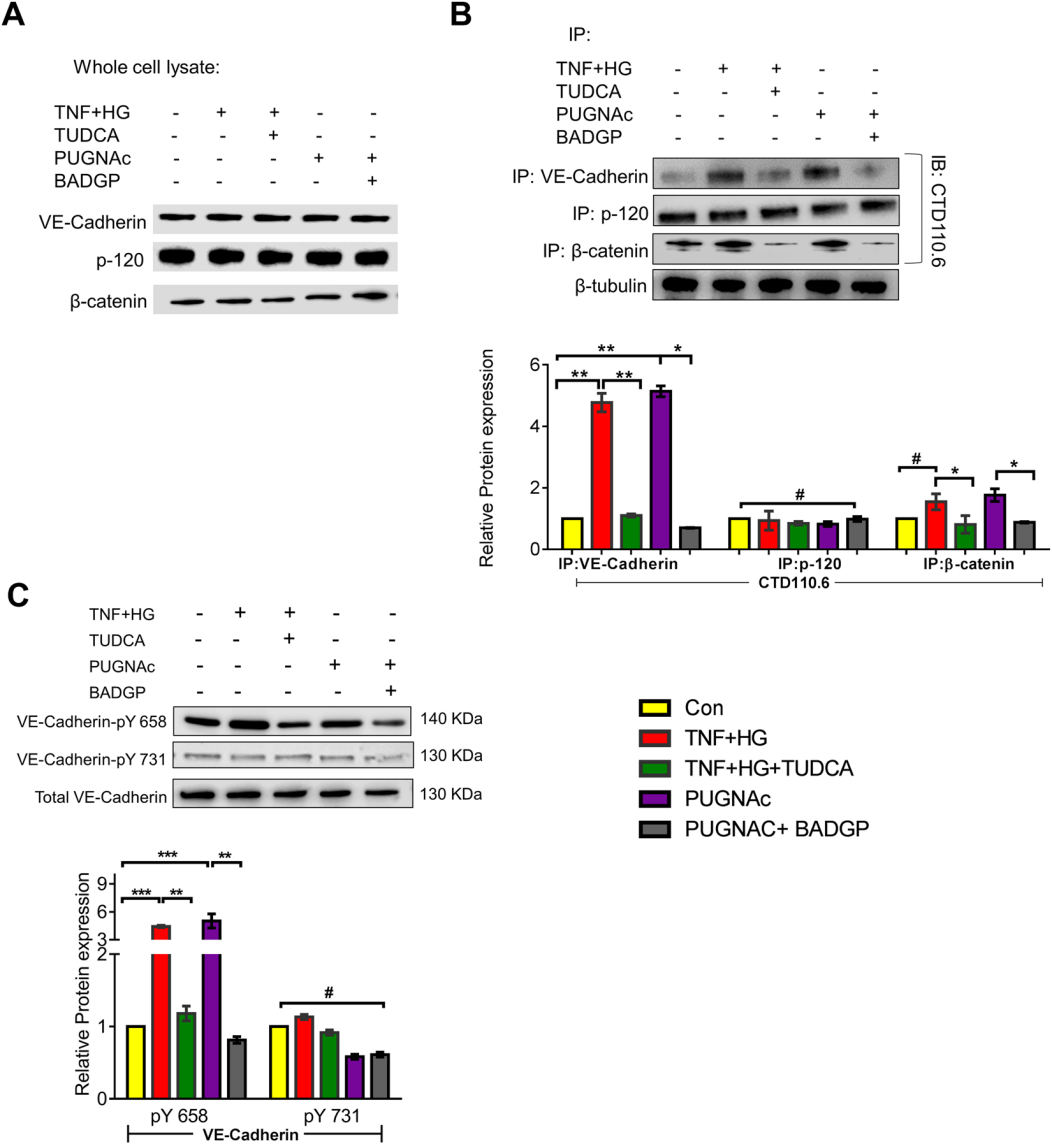
HRECs exposed to TNF-α, and high glucose for 24 h demonstrated alterations in VE-Cadherin and defective complex formation. The whole cell lysates were immunoprecipitated with indicated antibodies, and the immunocomplexes were analyzed for O-GlcNAcylation using anti-O-GlcNAc antibody (clone CTD110.6) (**A**). Whole cell lysates were loaded and probed for specific antibodies as loading controls. Immunoblots of phospho‐VE-Cadherin (pY658 and pY731) and total VE‐Cadherin from whole cell lysates (**B**). β-tubulin served as an internal control. Data represent Mean ± SEM from 3 independent experiments performed in duplicates. ***p < 0.001; **p < 0.01; *p < 0.05, ^#^p > 0.05.

Based on our previous observation that alterations in TER under the influence of TNF + HG treated HRECs coincided with a change in VE-Cadherin distribution^24^, we performed immunoprecipitation experiment to find whether O-GlcNAcylation of VE-Cadherin or its complex components β-catenin, p120 may lead to their dissociation from adherens junctions. The total cell lysate was immunoprecipitated with specific antibodies. The whole cell extracts were analyzed by respective immunoblots to indicate equal immunoprecipitation reactions (Fig. 6A). The immunocomplex was used to detect O-GlcNAcylation of VE-Cadherin or its complex partner’s p120 and β-catenin. While quiescent HREC monolayers demonstrated intact complex, HRECs exposed to TNF + HG demonstrated increased O-GlcNAcylated VE-Cadherin. Interestingly, only β-catenin but not p-120 was found to be O-GlcNAcylated. Based on these results, we then examined whether TUDCA was able to inhibit these effects. Indeed, TNF + HG induced O-GlcNAcylation of VE-Cadherin and β-catenin was prevented by TUDCA. Similar to TNF + HG, HRECs exposed to PUGNAc demonstrated a >2-fold increase in O-GlcNAcylated proteins with a significant reduction when co-incubated with BADGP.

### O-GlcNAcylation affects phosphorylation of VE-Cadherin

Tyrosine phosphorylation of the VE-Cadherin catenin complex has been reported to correlate with changes in the stability of VE-Cadherin adhesion in pro-inflammatory conditions^28^. Since glycosylation of proteins affects the phosphorylation of its proximal sites on proteins^29^, we checked the principal phosphorylation sites (Y658 and Y731) that is known to regulate barrier function^28^. As expected, HRECs exposed to TNF + HG demonstrated increased levels of pY658 levels in a manner similar to those cells treated with PUGNAc. Interestingly, HRECs pre-treated with TUDCA and challenged with TNF + HG demonstrated amelioration of phosphorylation of VE-Cadherin at tyrosine 658. While the O-glycosylation inhibitor (BADGP) reduced the pY658 levels of VE-Cadherin compared to PUGNAc, the levels of pY731 VE-Cadherin remained unaltered across all groups (Fig. 6B).

### GRP78 translocation to the plasma membrane interacts with VE-Cadherin

**Figure 7:**
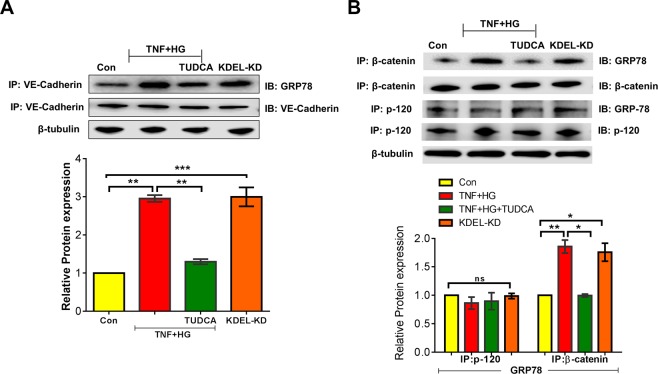
HRECs exposed to TNF-α, and high glucose for 24 h demonstrated alterations in VE-Cadherin and GRP78 association. Whole cell lysate were immunoprecipitated with antibody against VE‐Cadherin, and the immunocomplexes were analyzed by immunoblotting for GRP78 (**A**). Whole cell lysates were immunoprecipitated with complex partners (p-120 and β-catenin) and immunoblotted with GRP78 (**B**). β-tubulin served as an internal control. Data represent Mean ± SEM from 3 independent experiments performed in duplicates. ***p < 0.001; **p < 0.01.

We have shown that HRECs stimulated with TNF + HG increases the level of GRP78 protein and its translocation to the plasma membrane. We then examined the potential interaction of translocated GRP78 on the cell surface with VE-Cadherin. To this end, we immunoprecipitated VE-Cadherin from whole cell lysates and the immunocomplex was used to detect GRP78 protein expression. While quiescent HREC monolayers demonstrated no interaction, HRECs exposed to TNF + HG demonstrated the increased interaction of VE-Cadherin with GRP78, which interestingly, also reproduced with KDEL-KD cells. Based on these results, we then examined whether TUDCA was able to inhibit these effects. Indeed, TNF + HG induced interaction of GRP78 with VE-Cadherin was prevented by TUDCA (Fig. 7A). Additionally, we tested the interaction of GRP78 with other binding partners of VE-Cadherin by co-immunoprecipitation and found β-catenin but not p-120 to interact with GRP78 (Fig. 7B).

## Discussion

In this study, we report that chronic inflammatory activation of vascular endothelial cells and hyperglycemia promotes increased O-GlcNAcylation downstream of uncontrolled ER stress. In addition, we demonstrate that suppression of KDELR1 could enhance translocation of GRP78 from the ER to the plasma membrane. Given the emerging importance of cell surface GRP78 in controlling cell signaling, one of the most interesting observations in the current study is the interaction of translocated GRP78 with glycosylated VE-Cadherin present in the plasma membrane resulting in decreased TER of retinal endothelial cells. Interestingly, the afore-mentioned events abrogated with pre-incubation of endothelial cells with TUDCA, an ER stress inhibitor. Taken together with our previous observations, our results suggest endothelial activation mediated by chronic inflammation combined with hyperglycemia may result in a feed-forward loop of increased ER stress and changes in PTMs of proteins important for the harmony of endothelial junctions thus mediate endothelial permeability defects commonly observed in diabetes.

Several processes are linked to the pathogenesis of DR, including an imbalance in the retinal production of neuroprotective factors^30^, oxidative stress, inflammation, mitochondrial dysfunction, and ER stress^31^. We report here HRECs exposed to TNF-α and high glucose elicits a 3-6-fold increase in ER stress response. Upon ER stress, GRP78 is sequestered from the sensors and binds to unfolded/misfolded proteins to facilitate their refolding. The dissociation of GRP78 results in activation of the ER stress sensors and subsequently activates the UPR^32^. Experimental evidence suggests that all three UPR branches are activated in DR, contributing to retinal inflammation, apoptosis, and angiogenesis^12,33^. Previously we have identified IRE1α pathway activation in diabetic tie2-TNF mice, however we did not see any change in PERK and ATF6. Similarly, HRECs stimulated with TNF + HG treatment demonstrated activation of IRE1α pathway but not PERK and ATF6 suggesting that inflammation and hyperglycemia in endothelial cells results in diverse UPR pathways.

The recent discovery that ER stress not only induces the expression of ER chaperones to cope with stress but also actively promotes their surface translocation represents a paradigm shift for their functions^34^. Consistent with this notion, we report GRP78 translocation to the cell surface following TNF + HG treatment in human retinal endothelial cells. Interestingly, knockdown of KDELR1, a retrieval peptide that helps retain GRP78 in the cytosol also mimicked the effect. While the role of GRP78 as a pivotal regulator of the UPR is well established, the understanding of how translocated GRP78 regulates cell signaling is just emerging. While it is not necessary that translocation of GRP78 would lead to ER stress^18^, our data using ER stress inhibitor, TUDCA demonstrating a significant rescue in the GRP78 translocation suggests the two events are interrelated.

Glucose metabolism through the hexosamine biosynthesis pathway leads to the formation of uridine 5′-diphosphate-N-acetylglucosamine which serves as the substrate for PTM. O-GlcNAcylation regulates protein functions in response to nutrient availability, metabolic state, and cellular stress levels suggesting that any increase in O-GlcNAc could be considered as a general response to detrimental cellular conditions^35^. To this end using glycosylation array, we show several proteins being glycosylated in HRECs exposed to TNF + HG among which Focal adhesion kinase^25^ and Cathepsin D^26^ are some well-known players in vascular permeability were found to be upregulated. An elevated level of O-GlcNAcylation in endothelial cells exposed to TNF + HG are also reproducible with PUGNAc, a known inducer of O-GlcNAcylation. TNF + HG demonstrated an increase in OGT levels without altering OGA levels, point towards an increased O-GlcNAcylation of proteins under pro-inflammatory and hyperglycemic conditions. These results also indicate decreased OGA enzyme levels may lead to positive feedback in the endothelial cells under TNF + HG. This observation is in line with other animal and human subject studies that have shown excess O-GlcNAcylation linked to the pathogenesis of several diseases including diabetes^36–39^ and in the pathogenesis of DR^40^. Increased O-GlcNAcylation is reported under high glucose conditions in retinal vascular cells with pericytes being the most vulnerable^11^. To understand the role of O-GlcNAcylation in retinal endothelial cells better, we performed transendothelial migration assay with and without O-GlcNAcylation inhibitors. Our data not only suggest that ER stress induces transendothelial migration of leukocytes via upregulation of Icam1, Vcam1 and Ccl2 but it also involves O-glycosylation events suggesting a key role for PTMs in endothelial dysfunction.

Since PTMs could affect endothelial junction proteins we used immunoprecipitation and immunoblot methods to detect glycosylation of junction proteins. Interestingly, we found O-GlcNAcylation of VE-Cadherin, but not Zonula occludens-1 (ZO-1) and Junctional Adhesion molecule A (JAM-A) (Sup. Fig. 3). We have previously reported a loss in VE-Cadherin junctions by immunostaining in HRECs treated with TNF + HG^24^. Interestingly, in the current study, we showed that VE-Cadherin and its partner beta-catenin were, in fact, O-GlcNAcylated contributing to barrier dysfunction. Similar observation reported earlier in epithelial cadherin in cancer cells demonstrating O-GlcNAcylation of E-cadherin under ER stress affecting its trafficking^41^. Since the integrity of the VE-Cadherin and its partner’s p120 and β-catenin are critical for normal barrier function^42^, it is likely the explanation of increased vascular permeability in HRECs under the influence of TNF + HG. In addition to O-GlcNAcylation, other studies have reported tyrosine phosphorylation of VE-Cadherin and β-catenin by VEGF that results in alteration of vascular permeability as observed in DR and other conditions^43^. Taken together with these results, our data suggests that a variety of proteins are O-Glycosylated other than VE-Cadherin. Future studies beyond the scope of this study need to assess if VE-Cadherin alone or in consort with other proteins in HRECs play crucial role in the observed changes in permeability.

O-GlcNAc competes with phosphorylation at specific sites, and a reciprocal relationship between phosphorylation and O-GlcNAc has been observed globally^44,45^. This lead to the hypothesis that O-GlcNAcylation of VE-Cadherin may transiently block its phosphorylation. We observed increased levels of VE-Cadherin pY658 levels while pY731 remained unaltered in HRECs exposed to TNF + HG. Since pY685, but not pY731 has been shown to be crucial for the VE-Cadherin to stay in complex with its partners and actin cytoskeleton, it is unlikely pY731 plays a role in the O-Glycosylation events. Furthermore, our immunoprecipitation data also point to the fact that GRP78 cell surface translocation may affect VE-Cadherin by interacting with it and potentially contributing to the O-GlcNAcylation of VE-Cadherin and other junction proteins contributing to the observed loss of TER. Although our results support potential interplay between O-GlcNAcylation and phosphorylation of VE-Cadherin, the concerted action of both regulatory modifications remains to be determined. In support of our hypothesis, a recent study in endothelial cells suggested a relationship between O-GlcNAcylation and STAT3 phosphorylation under high glucose stress^46^.

TUDCA is an amphiphilic bile acid, and a taurine conjugate form of FDA approved ursodeoxycholic acid (UDCA). It is a well-known ER stress inhibitor^47^ and has been shown to protect against retinal damage^48,49^. Interestingly, our current studies at least in part ameliorate the ER stress and subsequent O-GlcNAcylation events suggesting some promising novel therapeutics for DR treatment. UDCA is an approved drug in China and is being tested and compared to TUDCA in a current US clinical trial (NCT02218619) for the treatment of New-Onset Type 1 Diabetes. Though UDCA has been shown to improve retinal vascular integrity in diabetic mice^50^, due to its poor absorption and extensive biotransformation the clinical effectiveness of UDCA may be limited^51^. The more hydrophilic TUDCA may be beneficial. However, the efficacy and safety of TUDCA in the treatment of DR are currently underexplored. Since repurposing drugs already approved by the regulatory authorities is an economical and time-efficient way to bring potential new therapies to patients quickly, we expect future studies will shed more light into the potential utility of TUDCA in the treatment of DR.

**Figure 8:**
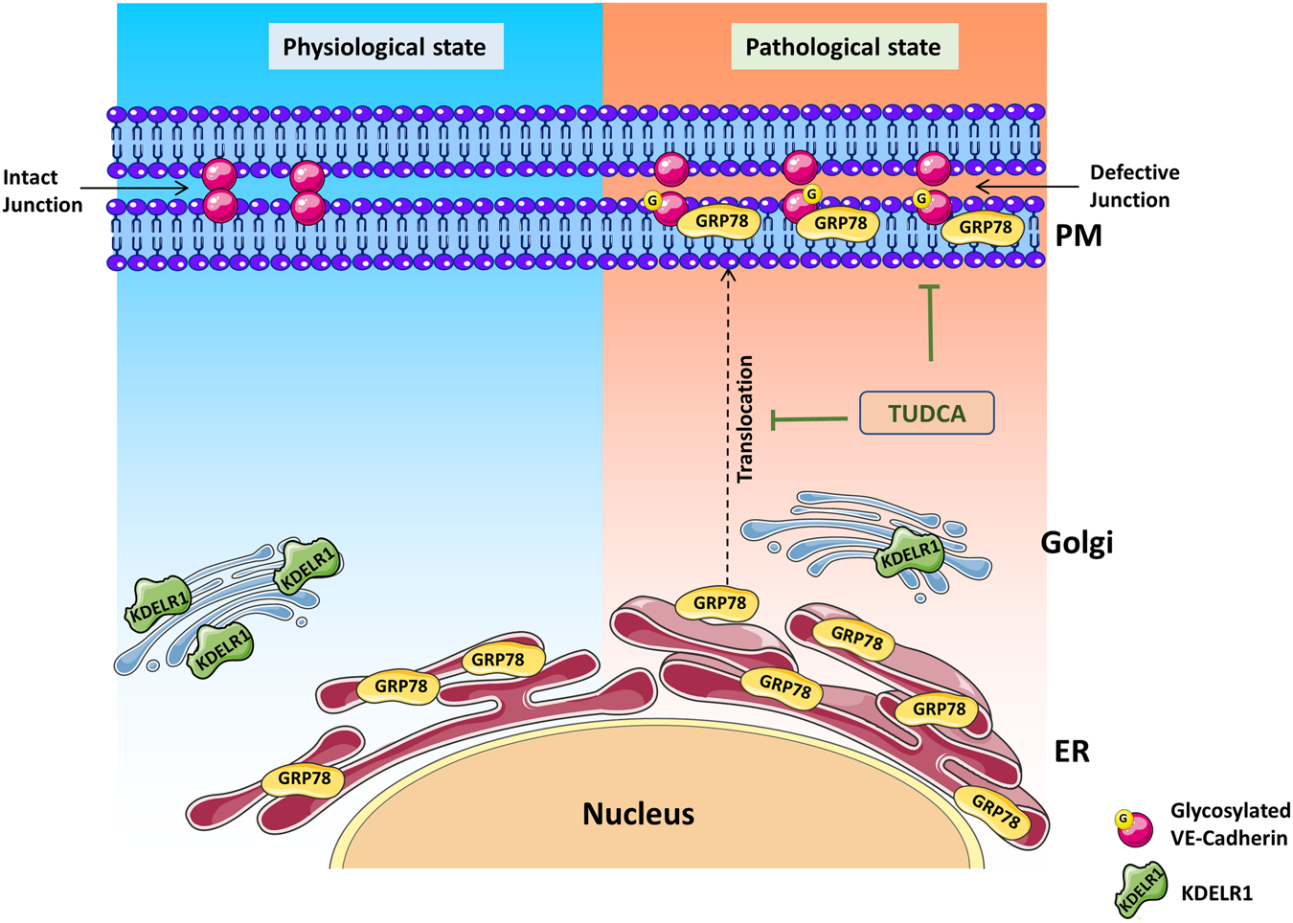
Schematic model of GRP78 translocation to the plasma membrane (PM) under ER stress leading to retinal endothelial permeability. In pathological state such as chronic inflammation and hyperglycemia will lead to up-regulation of ER stress in retinal endothelial cells. In the absence of KDELR-1, GRP78 translocates to the cell surface accentuating ER stress and O-GlcNAcylation of proteins. O-glycosylation of endothelial junction protein (VE-Cadherin) likely to form defective junctions leading to endothelial hyperpermeability. Inhibition of ER stress by tauroursodeoxycholic acid (TUDCA) protects against O-GlcNAcylation changes thus protects barrier integrity. Created with content from Servier Medical Art (https://smart.servier.com) under Creative Commons Attribution 3.0 Unported license (https://creativecommons.org/licenses/by/3.0/).

In conclusion, our studies using retinal endothelial cells subjected to hyperglycemia and inflammation demonstrate increased ER stress. Loss of linker peptide KDEL and subsequent cell surface translocation of GRP78 correlated with increased O-GlcNAcylation of endothelial junction proteins. Defective endothelial junctions further contribute to the loss of TER and likely result in increased vascular permeability. Interestingly, our results suggest that TUDCA is partially able to reverse these defects (Fig. 8). Our findings suggest an important role for ER stress and O-GlcNAcylation in altering the endothelial barrier function and reveal a potential therapeutic target in the treatment of DR.

## Materials and Methods

### Cell culture

Human retinal microvascular endothelial cells (HRECs; ACBRI-181) were obtained from Cell Systems (Kirkland, WA). Protocols and experiments involving human primary cells were conducted in accordance with The UTHSC Institutional Biosafety Committee guidelines following the NIH guidelines. Since the study involved de-identified cell lines from commercial sources, it is exempt from UTHSC Institutional Review Board review. HRECs were grown on attachment factor coated dishes (Gibco, Thermo Fisher Scientific, Waltham, MA) and cultured in EGM-2 basal media supplemented with EGM2-MV BulletKit (Lonza, Walkersville, MD). Cells between passage number 4 and 8 were used in all the experiments. To induce ER stress, cells were exposed to 20 ng/mL TNF-α (Sino Biological US, Inc., Wayne, PA) and 30 mM D-Glucose (Millipore Sigma, St Louis, MO) or 4 μM Tunicamycin (Millipore Sigma) for 24 h in serum-free media. L-Glucose (30 mM; Millipore Sigma) is an enantiomer of the more common D-Glucose served as control. To block ER stress, cells were pretreated with 20 μM TUDCA (Millipore Sigma) for 16 h. In glycosylation experiments, cells were exposed to 100 μM O-(2-acetamido-2-deoxy-D-glucopyranosylidene) amino-N-phenylcarbamate (PUGNAc, Millipore Sigma), a GlcNAc analogue that potently inhibits O-GlcNAcase in cells, leading to globally elevated levels of O-GlcNAcylation. To block glycosylation, cells were pre-treated with 100 μM benzyl-2-acetamido 2 deoxy α-D galactopyranoside (BADGP, Millipore Sigma).

### KDELR1 knockdown

HRECs at 60–80% confluence were used in the assay. Lipofectamine 2000 (Life Technologies, Thermo Fisher Scientific) and KDELR1 siRNA (30 pmol; Thermo Fisher Scientific) were diluted in OptiMEM (Gibco, Thermo Fisher Scientific) media, mixed and incubated for 5 min. The mixture was added to the cells, incubated for 6 h and replaced with a complete EGM-2MV medium for a further 48 h incubation. The cells were then used in subsequent experiments with various treatments.

### Cell fractionation

The cells were fractionated for membrane and cytosol fractions using ProteoExtract® Subcellular Proteome Extraction Kit per the manufacturer’s protocol (Millipore Sigma). After specific treatments, cells were washed and incubated cells with ice-cold Extraction I containing 5 μl of protease inhibitor mixture for 10 min at 4 °C with gentle agitation. To obtain the cell supernatant containing cytosol fraction, the above suspension was centrifuged at 1000 g at 4 °C for 10 min. The leftover pellet was resuspended in 1 mL of ice-cold Extraction II containing 5 μl of protease inhibitor mixture and incubated for 30 min at 4 °C. After centrifugation at 1000 g for 10 min at 4 °C, the supernatant (membrane/organelle fraction) was used to detect GRP78 protein expression.

### Gene expression analysis

Briefly, after various treatments, RNA was isolated using NucleoSpin^®^ RNA Plus kit (Macherey-Nagel GmbH, Takara Bio USA), following the manufacturer’s protocol. Subsequently, about 250 ng of total RNA from each sample was converted to cDNA using SuperScript III first-strand synthesis supermix (Thermo Fisher Scientific). The resulting cDNA sample served as a template for real-time qPCR using TaqMan probes (Table 1) and accompanying Master Mix (Applied Biosystems, Foster City, CA). PCR amplification was carried out using Quantstudio3 (Applied Biosystems) system with cycle conditions of the initial cycle: 50 °C for 2 min, and initial denaturation at 95 °C for 15 sec. This was followed by 40 cycles of denaturation at 95 °C for 15 sec, and annealing/extension of 60 °C for 1 min. The expression levels of target gene transcripts were determined using 2^−DDCt^ method and normalized to 18S rRNA.

**Table 1:** Taqman primers used in the study.

Genes	Assay ID	Reference Sequence	Amplicon length
18S ribosomal RNA (18s rRNA)	Hs03003631	NR_003286.4	69
heat shock protein 5 (Grp78)	Hs00607129	NM_005347.4	146
ER to nucleus signaling 1 (Ire1α)	Hs00176385	NM_001433.3	81
X-box binding protein 1 (Xbp1)	Hs03929085	NM_001079539.1	76
DNA-damage inducible transcript 3 (Chop)	Hs00358796	NM_001195053.1	93
Intercellular Adhesion molecule (Icam1)	Hs00164932	NM_000201.2	87
Vascular adhesion molecule (Vcam1)	Hs01003372	NM_001078.3	62
C-C motif chemokine ligand 2 (Ccl2)	Hs00234140	NM_002982.3	101

### Protein expression analysis

After specific treatments, cells were lysed using RIPA buffer [50 mM Tris-HCl (pH 8.0), 150 mM NaCl, 0.1% SDS, 0.2% sodium azide, 1% Triton X-100, 0.25% sodium deoxycholate, and 1x protease inhibitor]. Homogenate was further sonicated and incubated for 1 h on ice and centrifuged at 16,000 g for 5 min at 4 °C. The clear supernatant was collected and quantified for total protein by the BCA method. About 50 μg of protein was resolved on a NuPAGE Bis-Tris pre-cast gels (Thermo Fisher Scientific) and transferred to a nitrocellulose membrane. After blocking the membrane for an hour in 5% bovine serum albumin (BSA) in TBST, probed with appropriate primary antibodies (Table 2). This was followed by incubation with HRP-conjugated secondary antibodies and detection using an enhanced chemiluminescence kit (GE Healthcare, Chicago, IL). Targeted proteins were probed in separate blots. From independent experiments mean densitometry data were normalized to control using Image-J software and represented as the ratio of the target protein to β-tubulin or total protein of interest, where applicable.

**Table 2:** Antibodies used in the study.

Protein	Source	Part#
GRP-78	Thermo Fisher Scientific	PA1-014A
sXBP	Thermo Fisher Scientific	PA5-18940
CHOP	Thermo Fisher Scientific	MA1-250
β-Tubulin	Thermo Fisher Scientific	MA5-16308
O-GlcNAc (CTD110.6)	Santa Cruz Biotechnology	sc-59623
O-GlcNAc Transferase (OGT)	Sigma	O6264
O-GlcNAcase (OGA)	Sigma	SAB4200311
Phospho-VE-cadherin (Tyr731)	Thermo Fisher Scientific	44-1145G
Phospho -VE-cadherin (Tyr658)	Abcam	ab119785
VE-cadherin (Total)	Santa Cruz Biotechnology	sc-9989
KDELR1	Thermo Fisher Scientific	MA1-90944
NaKATPase	Abcam	ab7671
p-120	Abcam	ab72039
β-catenin	Abcam	ab32572
ZO-1	Santa Cruz Biotechnology	sc-33725
JAM-A	Santa Cruz Biotechnology	sc-53623

### Glycosylation Profiling

HREC cells exposed to TNF + HG for 24 h with and without TUDCA were lysed and shipped to Raybiotech for the assessment of glycosylation profile. Human Glycosylation Array 493 (Cat# GAH-GCM-493-4, Raybiotech) was used to detect glycosylation profiles of 493 human proteins, including, but not limited to, cytokines, chemokines, adipokines, growth factors, angiogenic factors, proteases, soluble receptors, soluble adhesion molecules. Capture antibodies without glycans for the 493 proteins printed onto glass slides were incubated with samples followed by through washing to remove unbound proteins. Subsequently, slides were incubated with five unique biotin-labeled lectins followed by Streptavidin-conjugated fluorescent dye (Cy3 equivalent) that recognizes the biotin attached to any bound lectin molecule. Finally, the glass slide was dried and laser fluorescence scanning was used to visualize the signals. Fluorescence signals were then compared to the array map to identify glycosylated proteins present in the samples.

### Immunoprecipitation

About 1.5 mg (~50 μl) of Protein G Dynabeads (Life Technologies, Thermo Fisher Scientific) were mixed with 10 μg of antibody in 200 μL PBS, incubated for 10–15 min at room temperature. Beads were washed once and proceeded for immunoprecipitation following the manufacturer’s instructions. Cell lysates (equivalent to 500 µg total protein) from control and each treatment was mixed with the Dynabead-Ab complex and incubated at 4 °C for overnight with slow rotation. The following day, beads were collected by magnetic separation. The supernatant was discarded, and the beads were washed twice with PBS. The immunocomplexes were boiled in sample buffer and resolved by NuPAGE pre-cast gels as described above and proceeded with immunoblot analysis.

### Immunocytochemistry

To reveal localization of GRP78 in the HREC cells, immunocytochemistry was performed. About 2 × 10^5^ cells were grown on 10 mm round coverslips placed in a 24-well plate, after specified treatments, were fixed with ice cold 100% methanol. Cells were labeled with Wheat germ agglutinin- AF 594 (Thermo Fisher Scientific, 5 μg/mL) for 10 min at room temperature followed by blocking in 5% normal goat serum containing 1% bovine serum albumin, 0.01% Triton-X for 30 min at room temperature. This was followed by staining for GRP78 (1:200) for 24 h at 4 °C. Thereafter, cells were washed with 0.1 M PBS containing 0.01% Triton-X and incubated with secondary antibody (goat anti-rabbit Alexa Fluor 488 (Thermo Fisher Scientific, 1:200). After nuclei were stained with DAPI, coverslips were mounted using the aqueous mounting medium (Lab Vision PermaFlour, Thermo Fisher Scientific) and were visualized using a Zeiss LSM 710 laser scanning confocal microscope with a 40x oil immersion objective. For immunostaining of VE-Cadherin, we followed our previously published methodology^24^.

### Retinal endothelial cell permeability *in vitro*

Measurements of trans-endothelial electrical resistance (TER) were performed using an electric cell-substrate impedance sensing (ECIS) device (ECIS Zθ; Applied Biophysics, Inc., Troy, NY), as described by us previously^24^. Briefly, HRECs were seeded at a density of 2 × 10^5^ cells/mL on gold electrodes (8W10E+; Applied Biophysics, Inc). After cells were grown for 16 h until maximum resistance was attained (∼1200 Ω), cells were exposed to various treatments, changes in resistance were monitored for up to 18 h. Resistance values for multiple wells, at 4000 Hz, were normalized to an identical starting resistance value, averaged and represented as normalized resistance over time.

### Transendothelial migration assay

Transendothelial migration of mononuclear leukocytes across the monolayer of HRECs was performed as described previously with slight modifications^27^. Briefly, HRECs were cultured in collagen coated transwell filter inserts (Corning Costar, Bodenheim, Germany) for 2 days. After specific treatments, the inserts were placed in fresh wells with serum free medium containing 1 × 10^6^ Dil labelled THP-1 (ATCC, TIB-202) cells added to the upper compartment. After 24 h, the number of transmigrated leukocytes was measured in the media from the bottom well by measuring the fluorescence intensity at an excitation of 549 and emission at 565 nm. Representative images of the transmigrated leukocytes were captured using EVOS fluorescence microscope.

### Statistical analysis

Data are presented as mean ± SEM of at least three independent experiments. For all quantitative experiments, statistical analyses were performed with a two-way ANOVA with Newmann-Keuls posthoc test for multiple group comparisons. For glycosylation profiling any ≥1.5-fold increase or ≤0.65-fold decrease in signal intensity for a single analyte between samples or groups were considered a measurable and significant difference in expression, provided that both sets of signals are well above background (Mean background + 2 standard deviations, accuracy ≈ 95%). A p-value of <0.05 was considered statistically significant. Data were analyzed for statistical interpretation using bioconductor limma statistical package or Prism 6 software; GraphPad Software, La Jolla, CA.

## Supplementary information

Supplementary Figures

## Data Availability

The datasets generated during and/or analyzed during the current study are available from the corresponding author on reasonable request.
