# Supplementary material for: GRP78 translocation to the cell surface and O-GlcNAcylation of VE-Cadherin contribute to ER stress-mediated endothelial permeability
Source: Sci Rep. 2019 Jul 25;9:10783. doi: 10.1038/s41598-019-47246-w (PMC6658495; doi:10.1038/s41598-019-47246-w)
Supplement: Supplementary file 1 — Supplementary Figures [file 41598_2019_47246_MOESM1_ESM.pdf]

GRP78 translocation to the cell surface and O-GlcNAcylation of VE-Cadherin contribute to ER stress-mediated endothelial permeability

Raji Lenin[1], Peter G Nagy[1], Kumar Abhiram Jha[1], Rajashekhar Gangaraju[1,2]*

[1] Department of Ophthalmology, University of Tennessee Health Science Center, Memphis, TN. USA.

[2] Department of Anatomy & Neurobiology, University of Tennessee Health Science Center, Memphis, TN. USA.

Supplementary Material

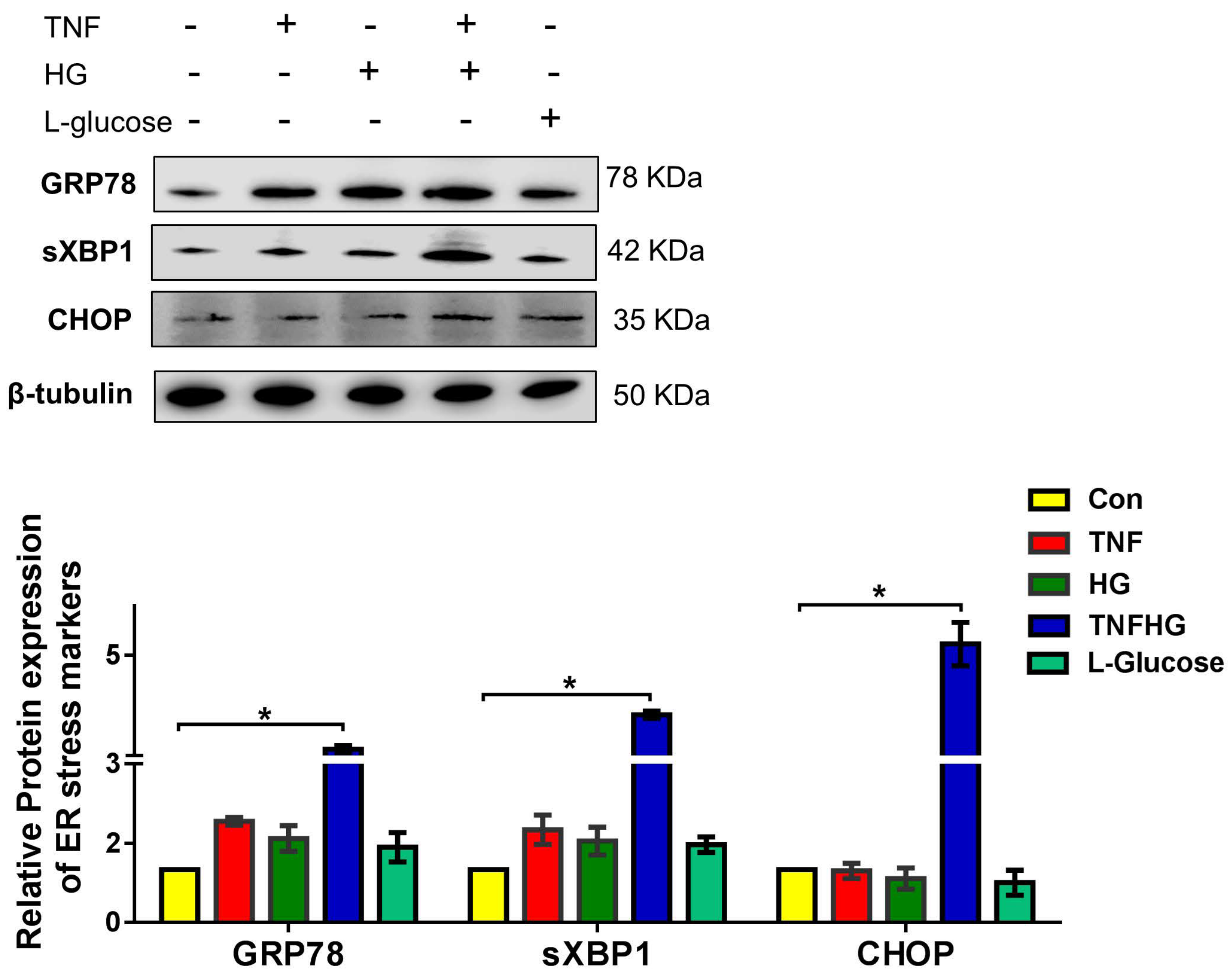

Sup. Fig. 1. HRECs exposed to the combination of TNFα and high glucose for 24 h demonstrated increased ER stress. Protein expression of ER stress markers with TNFα (TNF) alone or high glucose (HG) alone or the combination (TNF+HG). L-Glucose served as negative control for HG stress. Data represent Mean ± SEM from 3 independent experiments performed in duplicates. **p<0.01; *p<0.05; #p>0.05.

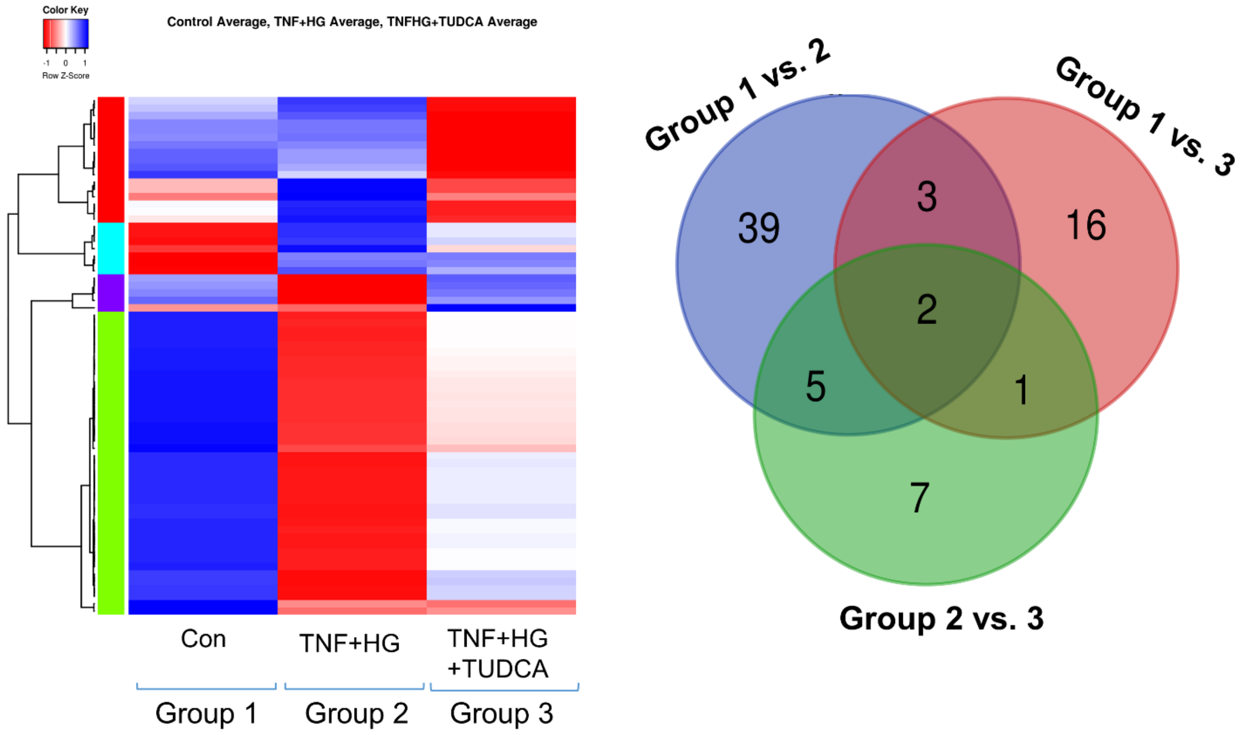

Sup. Fig. 2. Profile of various proteins glycosylated in the HREC cell lysates exposed to a combination of TNF+HG and TNF+HG/TUDCA compared to control. Left panel: heat map of glycosylated proteins relevant to inflammatory pathways differentially expressed between the various groups. A significant differential glycosylation with TNF+HG compared to control are partially normalized with TUDCA. Right Panel: Venn diagram showing the cluster of the proteins that are either >1.5 fold upregulated or <0.65 fold down regulated compared to other groups. See also Sup. Table 1. Data represent single experiment done in duplicates.

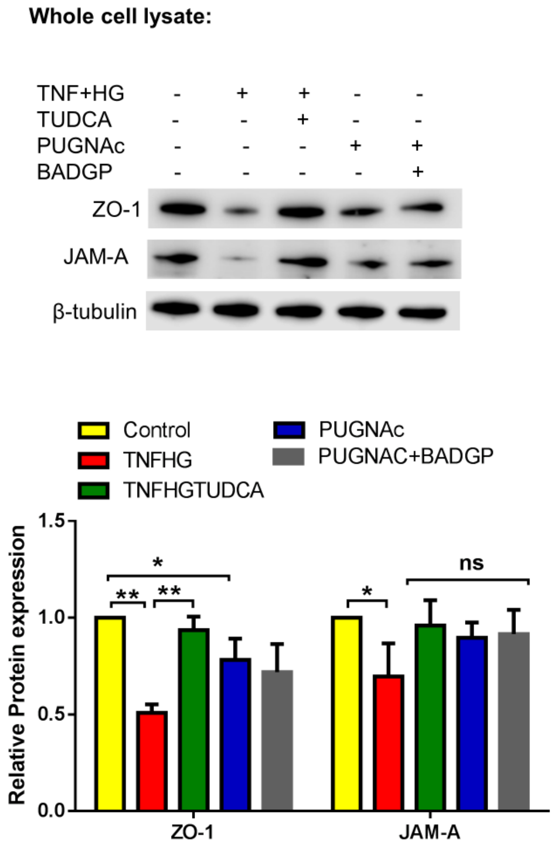

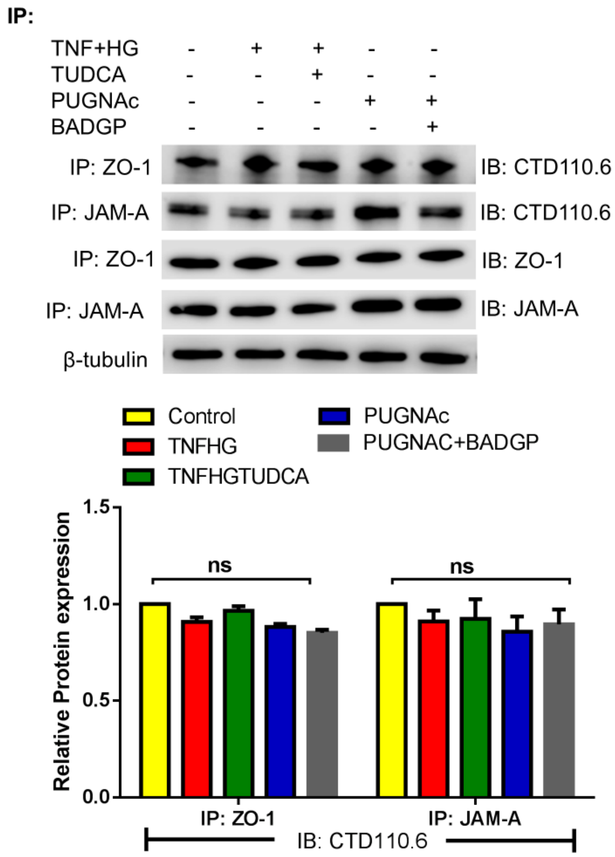

Sup. Fig. 3. HRECs exposed to TNFα, and high glucose for 24 h demonstrated alterations in ZO-1 and JAM-A. Left panel: Protein expression of ZO-1 and JAM-A. Right panel: The whole cell lysates were immunoprecipitated with indicated antibodies, and the immunocomplexes were analyzed for O-GlcNAcylation using anti-O-GlcNAc antibody (clone CTD110.6). Whole cell lysates were loaded and probed for specific antibodies for controls.

Sup. Table 1. The top 20 most significant changes in glycosylation profile.

Lenin et al					Top 20 Significant changes	
Protein	Control	TNF	TNF_TUDCA	TNF fold change vs. Control	Glycosylation state in TNF	TUDCA fold chan vs. TNF
"Fen 1 "	505.65	44.15	277.43	0.09	down regulated	6.28
"SART1 "	44.66	3.96	52.33	0.09	down regulated	13.20
"PSA-Free"	4045.82	376.27	1912.39	0.09	down regulated	5.08
"LH"	5102.85	532.15	3064.15	0.10	down regulated	5.76
"S-100b"	2026.92	230.14	1312.06	0.11	down regulated	5.70
"FGFR1 alpha "	3961.77	510.46	1837.51	0.13	down regulated	3.60
"ROCK1"	1498.90	206.34	725.47	0.14	down regulated	3.52
"CD71 "	3441.53	494.82	1677.52	0.14	down regulated	3.39
"Clusterin"	3849.67	565.88	2503.61	0.15	down regulated	4.42
"NR3C3"	4573.78	769.74	2882.99	0.17	down regulated	3.75
"GMNN "	6250.69	1058.14	4038.81	0.17	down regulated	3.82
"Kallikrein 2 "	3799.58	651.74	2298.33	0.17	down regulated	3.53
"MSHa"	5709.37	1005.50	3602.77	0.18	down regulated	3.58
"CRP"	1746.90	327.40	444.25	0.19	down regulated	1.36
"Procalcitonin"	3039.90	572.86	1899.22	0.19	down regulated	3.32
"Fyn"	2108.97	400.53	1574.22	0.19	down regulated	3.93
"Progesterone"	2443.79	469.43	1367.37	0.19	down regulated	2.91
"CA 15-3"	2157.72	438.39	1390.82	0.20	down regulated	3.17
"Nanog"	5024.06	1028.53	2824.05	0.20	down regulated	2.75
"IL-23p19"	2147.49	447.14	1103.21	0.21	down regulated	2.47
"ROR1"	1195.15	790.91	761.92	0.66	Unchanged	0.96
"LPS"	49.27	32.86	43.57	0.67	Unchanged	1.33
"ERRa"	718.56	527.86	742.68	0.73	Unchanged	1.41
"HSP90"	4126.40	3418.20	2079.01	0.83	Unchanged	0.61
"ADAMTS-13"	162.63	137.05	32.38	0.84	Unchanged	0.24
"OCT3/4"	243.71	218.59	441.18	0.90	Unchanged	2.02
"CRTAM"	913.02	824.89	294.76	0.90	Unchanged	0.36
"ApoE3"	663.11	618.67	349.56	0.93	Unchanged	0.57
"Chemerin"	1397.20	1375.48	871.62	0.98	Unchanged	0.63
"CNDP1"	1439.82	1443.28	1336.03	1.00	UP	0.93
"CD38"	1581.53	1608.41	1071.23	1.02	UP	0.67
"A2M"	728.76	745.17	516.14	1.02	UP	0.69
"B3GNT1"	1672.31	1789.61	1585.13	1.07	UP	0.89
"BAI-1 "	695.81	757.99	582.60	1.09	UP	0.77
"BLAME"	810.07	883.08	510.59	1.09	UP	0.58
"ApoD"	1183.29	1314.58	871.90	1.11	UP	0.66
"Cystatin C"	1811.30	2048.63	1568.96	1.13	UP	0.77
"Mesothelin"	600.01	717.76	667.84	1.20	UP	0.93
"APN"	956.74	1148.46	957.92	1.20	UP	0.83
"BMP-9"	1178.18	1452.56	1079.68	1.23	UP	0.74
"BNP "	723.54	907.12	829.56	1.25	UP	0.91
"AMICA"	734.67	1020.68	633.49	1.39	UP	0.62
"CD59"	493.78	818.79	701.93	1.66	UP	0.86
"DCBLD2"	636.21	1080.14	147.76	1.70	UP	0.14
"Cathepsin D"	921.19	1586.81	1563.81	1.72	UP	0.99
"DLL4"	576.07	1177.80	1180.36	2.04	UP	1.00
"FAK"	438.36	983.02	865.84	2.24	UP	0.88
"Fc RIIB/C"	284.41	698.51	434.03	2.46	UP	0.62
